# Increased levels of CSF total but not oligomeric or phosphorylated forms of alpha-synuclein in patients diagnosed with probable Alzheimer’s disease

**DOI:** 10.1038/srep40263

**Published:** 2017-01-10

**Authors:** Nour K. Majbour, Davide Chiasserini, Nishant N. Vaikath, Paolo Eusebi, Takahiko Tokuda, Wilma van de Berg, Lucilla Parnetti, Paolo Calabresi, Omar M. A. El-Agnaf

**Affiliations:** 1 Neurological Disorders Research Center, Qatar Biomedical Research Institute (QBRI), Hamad Bin Khalifa University (HBKU), Qatar Foundation, PO Box 5825, Doha, Qatar; 2 Department of Anatomy and Neurosciences, Neuroscience Campus Amsterdam, VU University Medical Centre, Amsterdam, The Netherlands; 3 Dipartimento di Medicina, sezione di Neurologia, Università degli Studi di Perugia, Perugia, Italy; 4 Neural Plasticity and Repair Unit, Department of Experimental Medical Sciences, Wallenberg Neuroscience Center, BMC A10, Lund University, Lund, Sweden; 5 Department of Neurology, Research Institute for Geriatrics, Kyoto Prefectural University of Medicine, Kyoto, 602-0841, Japan; 6 IRCCS Fondazione S. Lucia, Roma, Italy; 7 Life Sciences Division, College of Science and Engineering, Hamad Bin Khalifa University (HBKU), Education City, Qatar Foundation, PO Box 5825, Doha, Qatar

## Abstract

Several studies reported an association between CSF alpha-synuclein (α-syn) and tau in Alzheimer’s disease (AD), and demonstrated the significance of α-syn in improving the diagnostic sensitivity/specificity of classical AD CSF biomarkers. In the current study, we measured CSF levels of different α-syn species in a cohort of AD patients (n = 225) who showed a CSF profile typical of AD at baseline as well as in cognitively intact controls (n = 68). CSF total α-syn (t-α-syn) significantly increased in the AD group (p < 0.0001) compared to controls, while oligomeric- and phosphorylated-Ser129-α-syn did not change significantly. ROC analysis showed a sensitivity of 85% and a specificity of 84% (AUC = 0.88) in distinguishing AD from controls. T-α-syn levels correlated positively with tau species in AD group and negatively with baseline MMSE score. Our data support the added value of measurement of CSF α-syn species for further characterization of the CSF AD profile.

α-Synuclein (α-syn) is a pre-synaptic neuronal protein that has been linked to a number of neurodegenerative disorders named as “synucleinopathies”^1^. However, the role of α-syn in the pathogenesis of Alzheimer’s disease (AD) has been increasingly recognized since Uéda *et al*. reported the presence of a non-Aβ component in the extracellular plaques found in the brains of AD patients, which was shown to be a fragment of α-syn^2^. In fact, the role of α-syn became of particular interest when the co-existence of α-syn and tau pathology was observed in the brains of patients with AD, Parkinson’s disease (PD) and dementia with Lewy bodies (DLB)^3^. Although total tau (t-tau), phosphorylated tau (p-tau) and amyloid beta-42 (Aβ42) are state biomarkers of AD, some of the neuropathological changes observed in the brains of AD patients are not captured by these traditional biomarkers, such as α-syn inclusions “LBs”. Therefore, there is a need for additional biomarkers that can lead to better understanding and differential diagnosis. The aim of our study was to measure the levels of CSF α-syn species total- (t-), oligomeric- (o-) and phosphorylated-Ser129- (p-S129-) α-syn in a cohort of patients clinically diagnosed with probable AD who also exhibited a CSF profile positive for AD biomarkers, in support of the clinical diagnosis. To evaluate the diagnostic performance of CSF α-syn species in AD patients, we also included a control group composed of subjects who did not exhibit cognitive decline and who displayed CSF profiles negative for AD biomarkers. These were patients who had been diagnosed with other neurological diseases (OND). The correlation of CSF α-syn species with t-tau, p-tau and Aβ42 was also investigated.

## Results

### Patient characteristics

Demographic data and clinical features are reported in Table 1. The AD group had median disease duration of 2 years at the time of the lumbar puncture. No significant difference in the number of years of education was present. As expected, baseline MMSE scores were significantly lower in the AD group (*p* < 0.0001).

### Levels of α-syn species in the CSF of AD patients and control subjects

**Figure 1:**
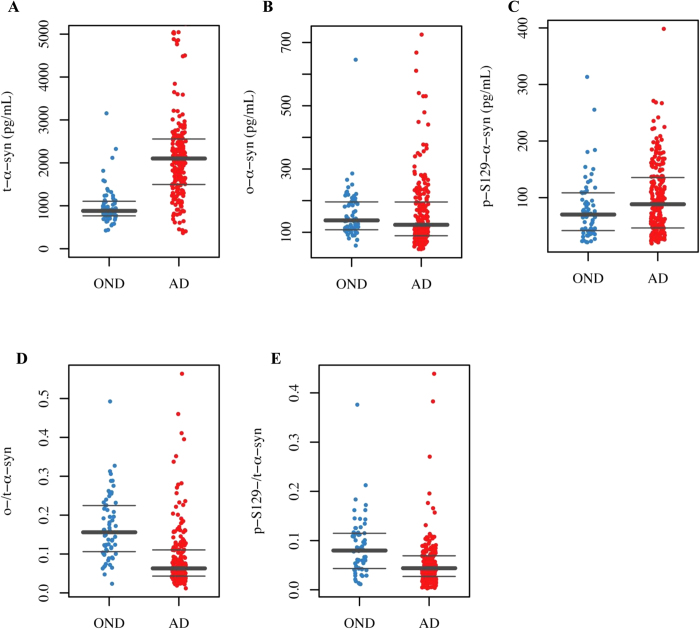
Boxplots of the values of CSF biomarkers observed in the AD and OND groups. Individual values of the CSF levels of **(A)** t-α-syn, **(B)** o-α-syn, and **(C)** p-S129-α-syn and the **(D)** o-/t-α-syn ratio and **(E)** p-S129-/t-α-syn ratio in patients with AD and OND. Horizontal bold lines indicate the medians; upper and lower horizontal lines indicate the range of values.

Due to the differences between the groups in some demographic and clinical parameters we used a logistic regression approach to assess the diagnostic performance of the α-syn species, adjusting for covariates such as sex, age and MMSE (Table 2). CSF t-α-syn levels were significantly higher in the AD group compared to the OND group (*p* < 0.0001, Fig. 1A), whereas CSF o-α-syn and p-S129-α-syn levels were similar in both diagnostic groups (*p* = 0.22 and *p* = 0.35, respectively, Fig. 1B,C). As a consequence, the o-/t-α-syn ratio and p-S129-/t-α-syn ratio were significantly lower in AD subjects compared to OND subjects (*p* < 0.001 and p < 0.0001 respectively, Fig. 1D,E).

**Figure 2:**
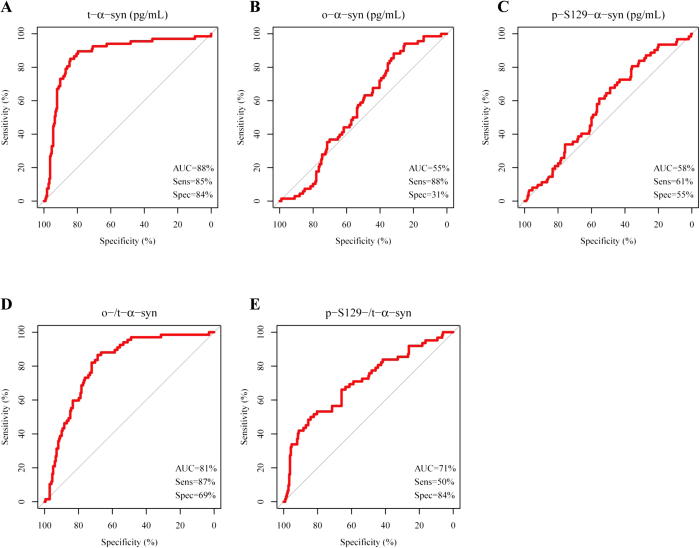
ROC curves of t-α-syn, o-α syn, p-S129-α-syn, the o-/t-α-syn ratio and the p-S129-/t-α-syn ratio and the fitted values of the multivariable logistic regression model.

ROC analysis was carried out for the α-syn species (Fig. 2), and t-α-syn achieved the best diagnostic performance compared to o-α-syn or p-S129-α-syn, with a sensitivity of 85% and a specificity of 84% (AUC = 0.87, 95% CI = 0.82–0.93, and cut-off = 1260 pg/ml). However, neither the o-/t-α-syn ratio nor the p-S129-/t-α-syn ratio improved the global diagnostic performance of t-α-syn in detecting AD (sensitivity = 87%, specificity = 79%, AUC = 0.82, 95% CI = 0.76–0.87, and cut-off = 11.5; sensitivity = 50%, specificity = 84%, AUC = 0.71, 95% CI = 0.63–0.78, and cut-off = 8.0, respectively).

### Correlation of CSF α-syn species with AD biomarkers and clinical features

**Figure 3:**
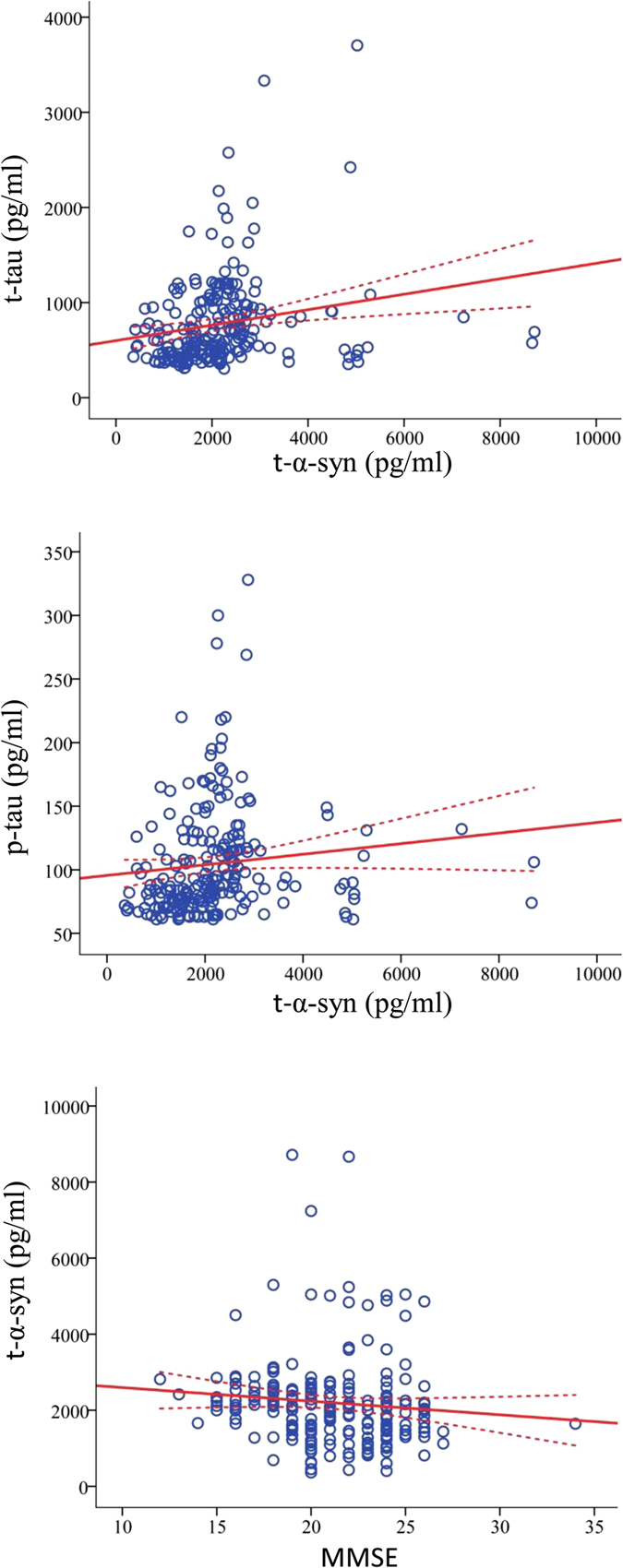
Scatter plots indicating the correlation between t-α-syn and t-tau, p-tau and MMSE scores. The correlation of CSF t-α-syn with t-tau (r = 0.31, *p* < 0.001), p-tau (r = 0.30, *p* < 0.001), and MMSE score (r = −0.21, *p* < 0.01) in the AD group **(A–C)**. The dotted line is the 95% prediction interval for the calculated regression line (solid line).

Correlation analysis of all the CSF biomarkers in the diagnostic groups is reported in Supplementary Table 1. As previously reported^4^, t-tau and p-tau CSF levels were significantly correlated in both diagnostic groups; however, the positive correlation was much stronger in the AD group. Interestingly, a significant positive correlation between t-α-syn and tau species was only noted in the AD group (r = 0.31, *p* < 0.001 for t-tau; r = 0.30, *p* < 0.001 for p-tau, Fig. 3A,B). Although similar correlations were observed within the OND group, they did not reach statistical significance. The levels of CSF o-α-syn positively correlated with t-α-syn levels (r = 0.20, *p* < 0.01). On the other hand, in both the AD and OND groups, no significant correlations between o- or p-S129-α-syn and AD CSF biomarkers were observed. The correlation analysis between the CSF biomarkers and clinical parameters in the AD and OND groups is reported in Supplementary Table 2. As expected, both t-tau and p-tau were negatively correlated with MMSE scores in the AD group. In addition, a significant inverse correlation between CSF t-α-syn levels and MMSE baseline scores was noted only within the AD group (r = −0.21, *p* < 0.01, Fig. 3C). No significant correlations were observed between the other α-syn species and age, education or disease duration in either diagnostic group.

## Discussion

The role of α-syn as a putative AD biomarker has been investigated in several studies, largely with contrasting results. Some studies have reported either unchanged or increased levels of t-α-syn in AD patients compared to various control groups (healthy or other neurological conditions)^5^,^6^,^7^.

In the present study, we used our newly developed, sensitive ELISAs to measure the concentration of t-α-syn and other pathologically important CSF α-syn species (o- and p-S129-α-syn), which are mainly associated with synucleinopathies. The CSF samples analyzed were selected from a large cohort of patients clinically diagnosed with probable AD who also had an AD-positive CSF biomarkers profile. As a control group, we included cognitively intact patients who had been diagnosed with other neurological diseases but had an AD-negative CSF biomarkers profile. Our main findings were: i) a significant increase in CSF t-α-syn levels in AD patients with respect to control subjects; ii) no significant change in CSF o- or p-S129-α-syn levels in AD patients; and iii) a significant correlation of t-α-syn levels with tau species and MMSE in the AD group.

The increase of t-α-syn in the AD group is in line with the findings of several previous reports^5^,^8^, but contrasts with others^9^,^10^. These discrepancies are likely due to several factors, including differences in the assay platform used, lack of control of contamination of the samples with red blood cells and heterogeneity of the patients included in the studies. In this study, we selected a cohort of AD patients by not only relying on an accurate clinical and neuropsychological evaluation but also choosing the patients according to the CSF profile of Aβ42, t-tau and p-tau, which further supports the initial AD diagnosis. Whereas, the OND group included cognitively normal subjects with CSF biomarker profile negative for AD, possibly excluding non-AD neurological disorders with neurodegeneration. This may explain the difference in diagnostic performance among the studies. Korff and colleagues found a sensitivity of 65% and a specificity of 74% in distinguishing AD patients from controls^11^, while in the present work, the performance of t-α-syn was better, reaching approximately 85% for both sensitivity and specificity.

A meaningful hypothesis about the increase of t-α-syn in the AD group relies on evidence of over-expression in the brain tissue of AD patients, as previously shown^12^, and/or on the neuronal damage related to AD. This, in turn, can increase the release of α-syn from damaged cells into the brain’s interstitial fluid and then into the CSF. This hypothesis is further supported by the significant positive correlation between t-α-syn and tau species we found in our cohort. This correlation agrees with previous reports^8^ and was observed solely in the AD group. Tau protein is considered the prototypical biomarker of neuronal damage because it is highly increased in the CSF of patients diagnosed with Creutzfeldt-Jakob disease^13^, a disease characterized by massive neurodegeneration, as well as in acute events such as traumatic brain injury^14^. Notably, CSF α-syn is also increased in these conditions and in other neurological diseases in which neurodegeneration is an important attribute^15^,^16^,^17^, further stressing the parallelism between tau and α-syn. In particular, the increase of α-syn in AD patients can be perceived as a marker of synapse loss and synapse disruption. It is worth noting that, consistent with other studies^11^,^18^, we observed a significant inverse correlation between the levels of CSF α-syn and cognitive function as measured by MMSE in our AD group.

The unique role of α-syn in AD pathology relative to synucleinopathies is also supported by the lack of a contribution of o- and p-S129-α-syn species to the diagnosis of AD. Substantial evidence suggests a neurotoxic role for o-α-syn in the pathogenesis of synucleinopathies, both *in vitro* and *in vivo*^19^, as α-syn oligomers are increased in brain homogenates and the CSF of PD and DLB patients^20^,^21^,^22^. Here, we found no significant differences in the level of o-α-syn in the AD group with respect to control subjects, thus confirming our previous reports^21^. Likewise, phosphorylated α-syn has been previously linked to PD pathogenesis, reflecting that most of the α-syn accumulated in LBs is phosphorylated at residue Ser129^23^,^24^. Elevated p-S129-/t-α-syn ratios have also been found in the CSF of PD patients^25^. In the current study, p-S129-α-syn did not show any significant change in our AD cohort, supporting the hypothesis that the oligomeric- and p-S129-α-syn species are intimately connected to the pathogenesis of synucleinopathies.

One possible limitation of this study is the lack of neuropathological assessment. It has been shown that AD biomarkers can detect the underlying AD pathology with high accuracy, but may not detect co-morbidity caused by the presence of other protein aggregates (Toledo *et al*. Acta Neuropathol, 2012)^5^. Co-morbidity may influence the accuracy of CSF biomarkers, including α-syn. Further studies should be devoted to the understanding of the complex relationships between CSF AD biomarkers and α-syn species across different types of dementia, possibly together with pathological confirmation. We also aim to include subjects with α-syn related dementias such as DLB and PD with dementia in future research to broaden our understanding of the potential role of α-syn as cognitive biomarkers.

In conclusion, our results in a well characterized cohort of AD patients and neurological controls further support the notion that t-α-syn most likely represents a marker of neuron loss and/or synaptic failure in AD. Future studies should be aimed at understanding the added values of α-syn species to the core AD biomarkers.

## Methods

### Patients and CSF sampling

The AD cohort enrolled in this study included patients who attended the Center for Memory Disturbances of the University of Perugia between 2008 and 2011 and underwent CSF analysis for diagnostic purposes after informed written consent. All of the patients underwent the following: a thorough clinical examination by experienced neurologists; a neuropsychological assessment, including the Mini Mental State Examination (MMSE); analysis of blood chemistry; and a brain CT and/or MRI scan to exclude major cerebrovascular diseases or other pathological brain conditions (e.g., hydrocephalus, tumors, hematomas, abscesses, etc.). The AD group was composed of 225 patients (M/F = 96/129) diagnosed with probable AD according to the research criteria proposed by Dubois *et al*.^26^ (M/F = 96/129). Each patient also had a CSF biomarker profile indicative of AD, according to the cut-offs used in our center (Aβ42 < 800 (pg/ml), t-tau > 300 pg/ml and p-tau > 60 (pg/ml)). As neurological controls, we selected 68 subjects who were admitted to the neurological ward and underwent CSF tapping for diagnostic reasons (e.g., headache, suspected myelopathy, etc.), were without clinical evidence of cognitive impairment and had CSF biomarkers profile negative for AD (other neurological diseases group, OND). In all subjects enrolled in this study, lumbar puncture was performed as a routine diagnostic procedure between 8:00 and 10:00 a.m. CSF (10 mL) was collected in sterile polypropylene tubes, centrifuged for 10 minutes at 2000 × g, divided into 0.5 mL aliquots and immediately frozen at −80 °C. CSF samples were collected according to a standard protocol following international guidelines^27^. The study was approved by the local Ethical Committee (Comitato Etico delle Aziende Sanitarie della Regione Umbria - CEAS Umbria) and informed written consent was signed by all patients enrolled or by their legal representatives. The work was carried out according to the Declaration of Helsinki.

### Immunoassays to quantify α-syn species in the CSF

CSF t-, o- and p-S129-α-syn levels were measured using our recently published ELISA assays^28^. Briefly, for measuring t-α-syn, a 384-well ELISA microplate was coated by overnight incubation at 4 °C with 0.1 μg/ml Syn-140 (sheep anti-α-syn polyclonal antibody) in 200 mM NaHCO_3_, pH 9.6 (50 μl/well). Similarly, Syn-140 was used for measuring p-S129-α-syn, while the conformation-specific monoclonal antibody (Syn-O2)^29^, which is specific for α-syn oligomers (0.2 μg/ml), was used as the primary antibody to capture o-α-syn. The plate was then washed with phosphate-buffered saline containing 0.05% Tween-20 (PBST) and incubated with 100 μl/well of blocking buffer (PBST containing 2.5% gelatin) for 2 hours at 37 °C. After washing, 50 μl of the CSF samples (thawed on ice and Tween-20 added to a final concentration of 0.05%) were added to each well, and the plate was incubated at 37 °C for another 2.5 hours. Antibodies included 11D12 (mouse anti-α-syn monoclonal antibody) for measuring t-α-syn, PS129 (mouse anti-pS129-α-syn monoclonal antibody) for measuring pS129-α-syn and FL-140 (rabbit polyclonal antibody, Santa Cruz Biotechnology, Santa Cruz, CA, USA) for measuring o-α-syn were diluted to the desired concentration (1:5000, 1:1,000 and 1:1,000, respectively) in the blocking buffer before being added to the corresponding wells and incubated at 37 °C for 2 hours. Next, the plate was washed and then incubated for 2 hours at 37 °C with 50 μl/well of species-appropriate secondary antibodies: donkey anti-mouse IgG HRP or goat anti-rabbit IgG HRP (Jackson ImmunoResearch, US), diluted in blocking buffer (1:20,000). After washing, the plate was incubated with 50 μl/well of an enhanced chemiluminescent substrate (SuperSignal ELISA Femto, Pierce Biotechnology, Rockford, IL). Then, the chemiluminescence (relative light units) was immediately measured using a VICTOR™ X3 multilabel plate reader (PerkinElmer). The standard curve for the ELISA assays was carried out using 50 μl/well of serial dilutions of recombinant human α-syn, p-S129-α-syn or o-α-syn in artificial CSF. The samples were screened in a blinded fashion and tested randomly. All the results were confirmed with at least two independent experiments. A series of internal controls was also run to check for run-to-run variations.

### Measurement of AD biomarkers

CSF Aβ42, total tau, and p-tau were measured using an ELISA technique (INNOTEST ß amyloid 1–42, hTAU-Ag, p-TAU 181 Ag, Fujirebio Europe, Gent, Belgium) as previously described^30^.

### Data analysis and statistics

Statistical analysis was performed using R software v. 2.15. Continuous variables were described by the median and interquartile range because data distributions were skewed. Correlations were calculated using Spearman’s Rho (r_s_). The Mann-Whitney test was used for initial comparisons between the two diagnostic groups (*p* < 0.05). The accuracy of the diagnostic value of the biomarkers^31^ was assessed by calculating the area under the curve (AUC) of the receiver operating characteristic (ROC) curve^32^. Cut-off values were calculated using sensitivity and specificity values that maximized Youden’s index. Because there was a significant difference in age (*p* < 0.0001) and in the distribution of gender (*p* < 0.01) between the groups, we corrected for these using a logistic regression approach to adjust for covariates. All CSF samples with an erythrocyte count >500 cells/μl were excluded from further analysis, as traces of blood may influence CSF α-syn levels^33^,^34^.

## Additional Information

**How to cite this article**: Majbour, N. K. *et al*. Increased levels of CSF total but not oligomeric or phosphorylated forms of alpha-synuclein in patients diagnosed with probable Alzheimer’s disease. *Sci. Rep.* **7**, 40263; doi: 10.1038/srep40263 (2017).

**Publisher's note:** Springer Nature remains neutral with regard to jurisdictional claims in published maps and institutional affiliations.

## Supplementary Material

Supplementary Data

## Figures and Tables

**Table 1:** Demographic data and clinical features of OND and AD subjects.

Demographics	OND	AD	p-values
	(n = 68)	(n = 225)	
Sex (M)	42 (62%)	96 (43%)	0.0094
Age (year)	63.7 ± 10.6	71.2 ± 8.8	<0.0001
Education (year)	9.1 ± 5.1	8.2 ± 4.5	0.3895
Disease years (year)	—	2.5 ± 1.4	—
MMSE	26.1 ± 3.2	21.1 ± 3.2	<0.0001

P-values and percentages are reported for sex and means, SD and p-values of the Mann-Whitney test are reported for the other variables.

**Table 2:** P-values of OND vs. AD comparisons of CSF biomarkers levels after adjustment for covariates (age, gender) using a regression model.

Biomarker	OND	AD	p-values
	(n = 68)	(n = 225)	
t-tau (pg/ml)	202.5 ± 55.5	776.1 ± 476.2	<0.0001
p-tau (pg/ml)	42.1 ± 9.5	105.5 ± 46.7	<0.0001
t-α-syn (pg/ml)	1119.0 ± 1114.4	2200.0 ± 1189.9	<0.0001
p-S129-α-syn (pg/ml)	81.8 ± 55.0	97.5 ± 61.8	0.346
o-α-syn (pg/ml)	158.5 ± 79.8	162.5 ± 113.3	0.2291
o-/t-α-syn	0.17 ± 0.08	0.09 ± 0.08	0.0003
p-S129-/t-α-syn	0.08 ± 0.06	0.04 ± 0.05	<0.0001

## References

[b1] Spillantini M. G. & Goedert M. The alpha-synucleinopathies: Parkinson’s disease, dementia with Lewy bodies, and multiple system atrophy. Ann N Y Acad Sci 920, 16–27 (2000). 11193145 10.1111/j.1749-6632.2000.tb06900.x

[b2] Uéda K. . Molecular cloning of cDNA encoding an unrecognized component of amyloid in Alzheimer disease. Proc Natl Acad Sci USA 90, 11282–11286 (1993). 8248242 10.1073/pnas.90.23.11282 PMC47966

[b3] Vekrellis K., Xilouri M., Emmanouilidou E., Rideout H. J. & Stefanis L. Pathological roles of α-synuclein in neurological disorders. Lancet Neurol 10, 1015–1025, doi: 10.1016/S1474-4422(11)70213-7 (2011). 22014436

[b4] Parnetti L. . Cerebrospinal fluid Tau/α-synuclein ratio in Parkinson’s disease and degenerative dementias. Mov Disord 26, 1428–1435, doi: 10.1002/mds.23670 (2011). 21469206

[b5] Toledo J. B., Korff A., Shaw L. M., Trojanowski J. Q. & Zhang J. CSF α-synuclein improves diagnostic and prognostic performance of CSF tau and Aβ in Alzheimer’s disease. Acta Neuropathol 126, 683–697, doi: 10.1007/s00401-013-1148-z (2013). 23812319 PMC3812407

[b6] Kasuga K. . Differential levels of alpha-synuclein, beta-amyloid42 and tau in CSF between patients with dementia with Lewy bodies and Alzheimer’s disease. J Neurol Neurosurg Psychiatry 81, 608–610, doi: 10.1136/jnnp.2009.197483 (2010). 20522869

[b7] Kapaki E., Paraskevas G. P., Emmanouilidou E. & Vekrellis K. The diagnostic value of CSF α-synuclein in the differential diagnosis of dementia with Lewy bodies vs. normal subjects and patients with Alzheimer’s disease. PLoS One 8, e81654, doi: 10.1371/journal.pone.0081654 (2013). 24282614 PMC3840054

[b8] Slaets S. . Increased CSF α-synuclein levels in Alzheimer’s disease: Correlation with tau levels. Alzheimers Dement, doi: 10.1016/j.jalz.2013.10.004 (2014). 24439167

[b9] Reesink F. E. . CSF alpha-synuclein does not discriminate dementia with Lewy bodies from Alzheimer’s disease. J Alzheimers Dis 22, 87–95, doi: 10.3233/jad-2010-100186 (2010). 20847452

[b10] Ohrfelt A. . Cerebrospinal fluid alpha-synuclein in neurodegenerative disorders-a marker of synapse loss? Neurosci Lett 450, 332–335, doi: 10.1016/j.neulet.2008.11.015 (2009). 19022350

[b11] Korff A. . α-Synuclein in cerebrospinal fluid of Alzheimer’s disease and mild cognitive impairment. J Alzheimers Dis 36, 679–688, doi: 10.3233/JAD-130458 (2013). 23603399 PMC3740054

[b12] Larson M. E. . Soluble α-synuclein is a novel modulator of Alzheimer’s disease pathophysiology. J Neurosci 32, 10253–10266, doi: 10.1523/JNEUROSCI.0581-12.2012 (2012). 22836259 PMC3425439

[b13] Skillbäck T. . Diagnostic performance of cerebrospinal fluid total tau and phosphorylated tau in Creutzfeldt-Jakob disease: results from the Swedish Mortality Registry. JAMA Neurol 71, 476–483, doi: 10.1001/jamaneurol.2013.6455 (2014). 24566866

[b14] Magnoni S. . Tau elevations in the brain extracellular space correlate with reduced amyloid-β levels and predict adverse clinical outcomes after severe traumatic brain injury. Brain 135, 1268–1280, doi: 10.1093/brain/awr286 (2012). 22116192 PMC3326246

[b15] Blennow K., Hampel H., Weiner M. & Zetterberg H. Cerebrospinal fluid and plasma biomarkers in Alzheimer disease. Nat Rev Neurol 6, 131–144, doi: 10.1038/nrneurol.2010.4 (2010). 20157306

[b16] Wang H. . Cerebrospinal fluid α-synuclein levels are elevated in multiple sclerosis and neuromyelitis optica patients during replase. J Neurochem 122, 19–23, doi: 10.1111/j.1471-4159.2012.07749.x (2012). 22469018

[b17] Kasai T. . Increased α-synuclein levels in the cerebrospinal fluid of patients with Creutzfeldt-Jakob disease. J Neurol 261, 1203–1209, doi: 10.1007/s00415-014-7334-7 (2014). 24737170

[b18] Larson M. E. . In J Neurosci Vol. 32 10253–10266 (2012). 10.1523/JNEUROSCI.0581-12.2012 PMC3425439 22836259

[b19] Winner B. . *In vivo* demonstration that alpha-synuclein oligomers are toxic. Proc Natl Acad Sci USA 108, 4194–4199, doi: 10.1073/pnas.1100976108 (2011). 21325059 PMC3053976

[b20] Tokuda T. . Detection of elevated levels of α-synuclein oligomers in CSF from patients with Parkinson disease. Neurology 75, 1766–1772, doi: 10.1212/WNL.0b013e3181fd613b (2010). 20962290

[b21] Hansson O. . Levels of cerebrospinal fluid α-synuclein oligomers are increased in Parkinson’s disease with dementia and dementia with Lewy bodies compared to Alzheimer’s disease. Alzheimers Res Ther 6, 25, doi: 10.1186/alzrt255 (2014). 24987465 PMC4075410

[b22] Paleologou K. E. . Detection of elevated levels of soluble alpha-synuclein oligomers in post-mortem brain extracts from patients with dementia with Lewy bodies. Brain 132, 1093–1101, doi: 10.1093/brain/awn349 (2009). 19155272

[b23] Anderson J. P. . Phosphorylation of Ser-129 is the dominant pathological modification of alpha-synuclein in familial and sporadic Lewy body disease. J Biol Chem 281, 29739–29752, doi: 10.1074/jbc.M600933200 (2006). 16847063

[b24] Fujiwara H. . alpha-Synuclein is phosphorylated in synucleinopathy lesions. Nat Cell Biol 4, 160–164, doi: 10.1038/ncb748 (2002). 11813001

[b25] Wang Y. . Phosphorylated α-synuclein in Parkinson’s disease. Sci Transl Med 4, 121ra120, doi: 10.1126/scitranslmed.3002566 (2012). PMC3302662 22344688

[b26] Dubois B. . Research criteria for the diagnosis of Alzheimer’s disease: revising the NINCDS-ADRDA criteria. Lancet Neurol 6, 734–746, doi: 10.1016/S1474-4422(07)70178-3 (2007). 17616482

[b27] Teunissen C. E. . A consensus protocol for the standardization of cerebrospinal fluid collection and biobanking. Neurology 73, 1914–1922, doi: 10.1212/WNL.0b013e3181c47cc2 (2009). 19949037 PMC2839806

[b28] Majbour N. K. . Oligomeric and phosphorylated alpha-synuclein as potential CSF biomarkers for Parkinson’s disease. Mol Neurodegener 11, 7, doi: 10.1186/s13024-016-0072-9 (2016). 26782965 PMC4717559

[b29] Vaikath N. N. . Generation and characterization of novel conformation-specific monoclonal antibodies for α-synuclein pathology. Neurobiol Dis 79, 81–99, doi: 10.1016/j.nbd.2015.04.009 (2015). 25937088

[b30] Parnetti L. . Performance of aβ1-40, aβ1-42, total tau, and phosphorylated tau as predictors of dementia in a cohort of patients with mild cognitive impairment. J Alzheimers Dis 29, 229–238, doi: 10.3233/JAD-2011-111349 (2012). 22232006

[b31] Eusebi P. Diagnostic accuracy measures. Cerebrovasc Dis 36, 267–272, doi: 10.1159/000353863 (2013). 24135733

[b32] Robin X. . pROC: an open-source package for R and S+ to analyze and compare ROC curves. BMC Bioinformatics 12, 77, doi: 10.1186/1471-2105-12-77 (2011). 21414208 PMC3068975

[b33] Hong Z. . DJ-1 and alpha-synuclein in human cerebrospinal fluid as biomarkers of Parkinson’s disease. Brain 133, 713–726, doi: 10.1093/brain/awq008 (2010). 20157014 PMC2842513

[b34] Barbour R. . Red blood cells are the major source of alpha-synuclein in blood. Neurodegener Dis 5, 55–59, doi: 10.1159/000112832 (2008). 18182779

