# Supplementary material for: Increased levels of CSF total but not oligomeric or phosphorylated forms of alpha-synuclein in patients diagnosed with probable Alzheimer’s disease
Source: Sci Rep. 2017 Jan 10;7:40263. doi: 10.1038/srep40263 (PMC5223278; doi:10.1038/srep40263)
Supplement: Supplementary Data [file srep40263-s1.pdf]

Increased levels of CSF total but not oligomeric or phosphorylated forms of alpha-synuclein in patients diagnosed with probable Alzheimer's disease

Nour K. Majbour, MSc[1, 2†], Davide Chiasserini, PhD[3†], Nishant N. Vaikath, MSc[1, 4], Paolo Eusebi, PhD[3], Takahiko Tokuda, MD, PhD[5], Wilma van de Berg, PhD[2], Lucilla Parnetti, MD, PhD[3,] Paolo Calabresi, MD[3, 6], Omar M.A. El-Agnaf, PhD[1,7]

[1]*Neurological Disorders Research Center, Qatar Biomedical Research Institute (QBRI), Hamad Bin Khalifa University (HBKU), Qatar Foundation, PO Box 5825, Doha, Qatar*

[2]*Department of Anatomy and Neurosciences, Neuroscience Campus Amsterdam, VU University Medical Centre, Amsterdam, The Netherlands.*

[3]*Dipartimento di Medicina, sezione di Neurologia, Università degli Studi di Perugia, Perugia, Italy*

[4]*Neural Plasticity and Repair Unit, Department of Experimental Medical Sciences, Wallenberg Neuroscience Center, BMC A10, Lund University, Lund, Sweden*

[5]*Department of Neurology, Research Institute for Geriatrics, Kyoto Prefectural University of Medicine, Kyoto, 602-0841, Japan*

[6]*IRCCS Fondazione S. Lucia, Roma, Italy*

[7]*Life Sciences Division, College of Science and Engineering, Hamad Bin Khalifa University (HBKU), Education City, Qatar Foundation, PO Box 5825, Doha, Qatar*

[†]These authors contributed equally to this work.

Supplementary Table 1

Spearman correlations between CSF biomarkers in OND and AD groups (*p <0.05, **p <0.01, ***p <0.001).

		$A\beta_{42}$	t-Tau	p-Tau	t-α-syn	p-S129-α-syn	o-α-syn	o-/t-α-syn	p-S129-/t-α-syn
**OND**	$A\beta_{42}$	1.00							
	t-Tau	0.10	1.00						
	p-Tau	0.07	0.47***	1.00					
	t-α-syn	-0.09	0.13	0.14	1.00				
	p-S129-α-syn	-0.13	-0.14	-0.29	0.15	1.00			
	o-α-syn	-0.12	-0.10	-0.19	0.03	-0.02	1.00		
	o-/t-α-syn	-0.05	0.08	0.16	-	0.11	-	1.00	
	p-S129-/t-α-syn	-0.10	-0.18	-0.30*	-	-	0.84***		1.00
**AD**	$A\beta_{42}$	1.00							
	t-Tau	0.00	1.00						
	p-Tau	0.06	0.72***	1.00					
	t-α-syn	0.10	0.31***	0.30***	1.00				
	p-S129-α-syn	0.02	0.12	0.13	0.12	1.00			
	o-α-syn	0.00	-0.10	-0.01	0.20**	0.03	1.00		
	o-/t-α-syn	0.10	0.26	0.22**	-	0.04	-	1.00	
	p-S129/t-α-syn	-0.05	-0.04	-0.04	-	-	0.81***	-	1.00

Supplementary Table 2

Spearman correlations between CSF biomarkers, age, education and MMSE in OND and AD groups (*p <0.05, **p <0.01, ***p <0.001).

		Age	Education	Disease years	MMSE
**OND**	$A\beta_{42}$	-0.20	-0.28	0.52*	0.29
	t-Tau	0.35	-0.17	-0.38	-0.38*
	p-Tau	-0.07	-0.19	-0.18	-0.08
	t-α-syn	0.03	0.01	-0.24	-0.01
	p-S129-α-syn	0.00	0.24	-0.15	0.00
	o-α-syn	-0.04	0.17	-0.43	-0.13
	o-/t-α-syn	-0.05	-0.10	-0.01	0.15
	p-S129-/t-α-syn	0.03	0.28	0.23	0.07
**AD**	$A\beta_{42}$	0.21**	-0.17	0.12	-0.08
	t-Tau	-0.04	0.18	0.10	-0.47***
	p-Tau	-0.15	0.18	0.10	-0.65***
	t-α-syn	0.14	-0.02	0.11	-0.21**
	p-S129-α-syn	0.03	-0.02	0.09	-0.06
	o-α-syn	-0.09	0.11	0.03	0.01
	o-/t-α-syn	0.13	-0.13	0.01	-0.14
	p-S129-/t-α-syn	-0.05	0.04	0.01	0.04
